# Waterproof coatings for high-power laser cavities

**DOI:** 10.1038/s41377-018-0118-6

**Published:** 2019-01-23

**Authors:** Xinbin Cheng, Siyu Dong, Song Zhi, Sebastian Paschel, Istvan Balasa, Detlev Ristau, Zhanshan Wang

**Affiliations:** 1 MOE Key Laboratory of Advanced Micro-Structured Materials, Shanghai, 200092 China; 2 Institute of Precision Optical Engineering, School of Physics Science and Engineering, Tongji University, Shanghai, 200092 China; 3 IFSA Collaborative Innovation Center, Shanghai Jiao Tong University, Shanghai, 200240 China; 4 Laser Zentrum Hannover e.V., Laser Components Department, Hannover, 30419 Germany

**Keywords:** Applied optics, Optical materials and structures

## Abstract

With the ever-increasing laser power and repetition rate, thermal control of laser media is becoming increasingly important. Except for widely used air cooling or a bonded heat sink, water cooling of a laser medium is more effective in removing waste heat. However, how to protect deliquescent laser media from water erosion is a challenging issue. Here, novel waterproof coatings were proposed to shield Nd:Glass from water erosion. After clarifying the dependence of the waterproof property of single layers on their microstructures and pore characteristics, nanocomposites that dope SiO_2_ in HfO_2_ were synthesized using an ion-assisted co-evaporation process to solve the issue of a lack of a high-index material that simultaneously has a dense amorphous microstructure and wide bandgap. Hf_0.7_Si_0.3_O_2_/SiO_2_ multifunctional coatings were finally shown to possess an excellent waterproof property, high laser-induced damage threshold (LIDT) and good spectral performance, which can be used as the enabling components for thermal control in high-power laser cavities.

Although hydrophobic coatings have been well studied regarding their water repellency or moisture proof property^1–3^, they are not applicable under cooling water conditions^4–9^, where coatings are immersed in water over long periods of time. For waterproof coatings, Murahara has proposed a hard-water-resistant coating that can protect KDP from dissolving^10^. However, his approach was limited to the use of SiO_2_ single layers and failed to meet the requirement of multilayers in which both high- and low-index materials are needed^11,12^. Although the microstructures of widely used optical coatings prepared using physical vapor deposition techniques have been extensively studied regarding stress, the refractive index, humidity-induced spectral shift and so on^13–15^, the correlation between the coating microstructure and waterproof property has not been specifically addressed. To meet the challenging requirement of waterproof cavity mirrors, the dependence of water resistance on the microstructure of single- and multilayers was studied first. On the basis of these results, microstructure and bandgap engineering were performed to synthesize nanocomposite-based cavity mirrors with exceptional multifunctionality, which has been previously unattainable. The proposed multifunctional coating can be used as an enabling technology to realize high-repetition-rate laser inertial confinement fusion, which can then be used as a fusion power plant.

The schematic of a water-cooled Nd:Glass laser cavity is shown in Fig. 1. SiO_2_ and HfO_2_ are the dominant low and high index materials for the near-infrared region^16,17^. The water resistance of single layers was investigated first. SiO_2_ and HfO_2_ single layers were prepared using electron beam evaporation (EBE) and ion assisted deposition (IAD) processes, respectively. The coatings prepared by the EBE process had a porous microstructure and failed to protect Nd:Glass from water erosion. The SiO_2_ layers peeled off of Nd:Glass substrates with heating in a water bath over a period of several hours. The eroded morphologies of the SiO_2_ layer were recorded using a camera and an optical microscope, as shown in Fig. 2a1, a2. A cross-sectional transmission electron microscopy (TEM) image of a Ta_2_O_5_ coating was used instead to reveal the microstructure of the SiO_2_ coating, where the brighter areas represent pores. It can be seen that an abundance of pores exist between the columns, which are open, elongated, and oriented perpendicular to the coating surface, as shown in Fig. 2a3. Water can quickly penetrate into the Nd:Glass surface through these open microscopic channels by capillarity, which is schematically illustrated in Fig. 2a4. Erosion of the Nd:Glass surface led to the delamination of SiO_2_ coatings. For porous HfO_2_ layers on Nd:Glass substrates, visible damage was observed after testing the samples in a hot water bath for several days, as shown in Fig. 2b1, b2. The relatively longer survival time of these layers is attributed to their different microstructure and pore characteristics. Figure 2b3 shows that there is a transition from an amorphous microstructure to a polycrystalline microstructure as the coating grows thicker. Compared to the elongated pores in the amorphous coatings, the polycrystalline microstructure in the upper part of the HfO_2_ coating results in a substantially lower number of long and open pores. Water diffusion through the HfO_2_ layer is delayed due to these crisscrossing channels, which are illustrated in Fig. 2b4. This comparison shows that for porous coatings, the polycrystalline microstructure offers advantages with respect to water resistant compared to an amorphous microstructure.

**Fig. 1:**
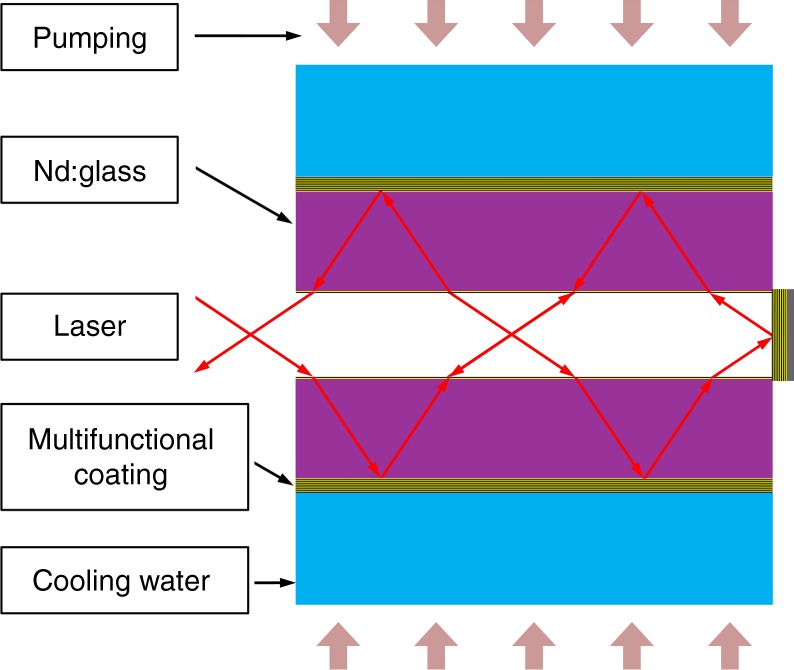
Schematic of a water-cooled Nd:Glass laser cavity

**Fig. 2:**
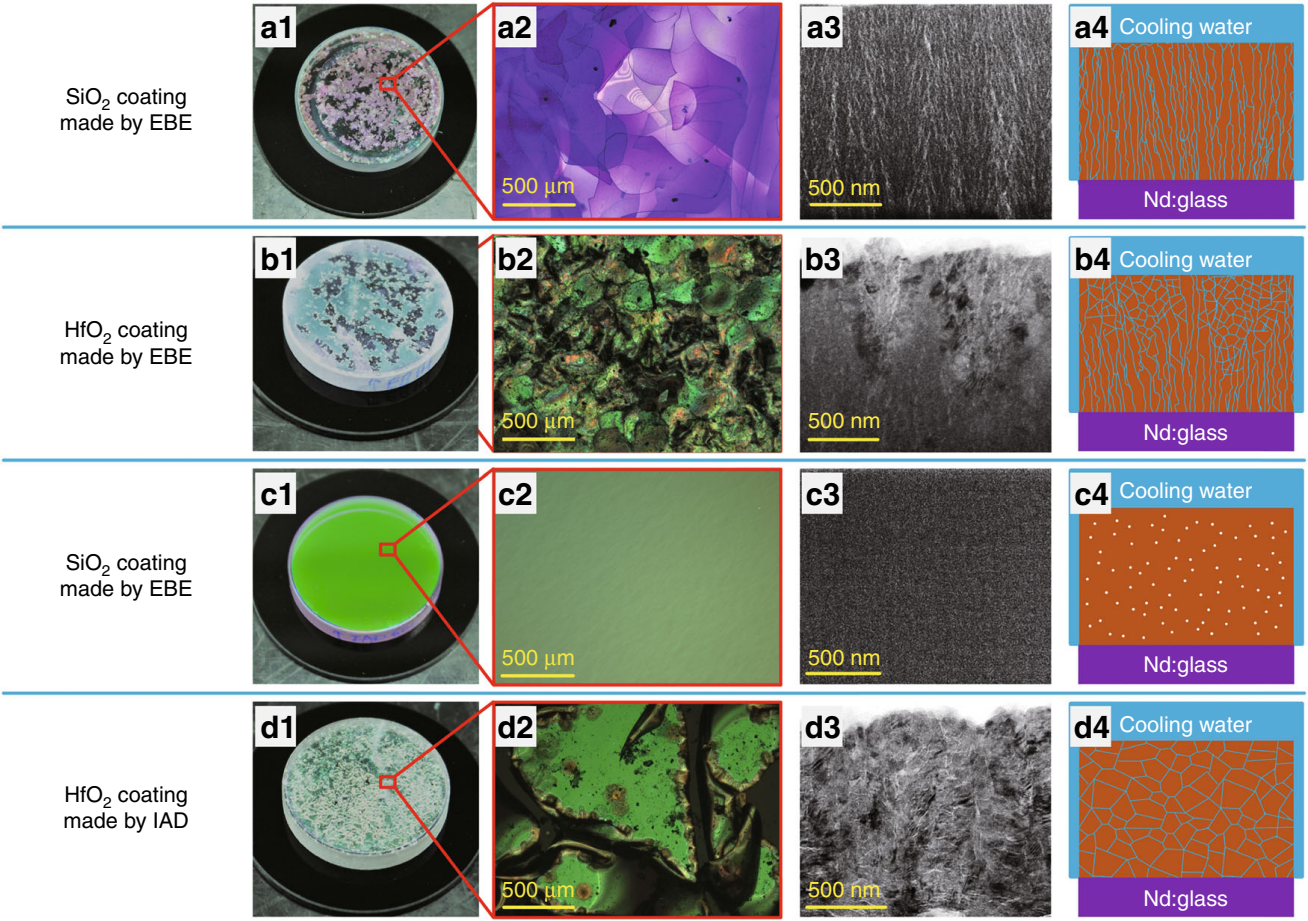
SiO_2_ and HfO_2_ coatings prepared by EBE and IAD processes. a1–d1 Optical photographs, a2–d2 microscopic images, a3–d3 cross-sectional TEM images, a4–d4 schematics of water diffusion along the nanopores in the coatings. It is worth noting that the TEM images of the SiO_2_ coatings are replaced using TEM images of Ta_2_O_5_ coatings to better reveal the microstructure. TEM images and the electron diffraction pattern of the SiO_2_ coatings are shown in Figure S2 and S3 in the Supplementary Information

IAD can significantly reduce the number of pores in coatings via energetic ion bombardment during film growth. The densified SiO_2_ layers did not peel off of Nd:Glass substrates with water bath heating for several months, as shown in Fig. 2c1, c2. Although no visible pores can be identified in the cross-sectional TEM image in Fig. 2c3, some nanometer-sized closed pores are assumed to be embedded in the SiO_2_ coatings^18^. These closed nanopores are isolated from each other, as schematically presented in Fig. 2c4. There are no channels for water diffusion in dense amorphous SiO_2_ coatings, so Nd:Glass substrates can be protected from corrosion. HfO_2_ coatings prepared by the IAD process exhibited a polycrystalline microstructure. Although these coatings were also very dense, they failed to protect Nd:Glass from water erosion. Figure 2d1, d2 shows that HfO_2_ coatings were severely damaged after immersion in a hot water bath for several weeks. The cross-sectional TEM image shown in Fig. 2d3 shows an abundance of complex grain boundaries with nanopores. These nanopores are connected with each other, resulting in a network of zigzag channels from which water can find ways to reach and erode the Nd:Glass, as shown in Fig. 2d4. This comparison reveals that for dense single layers, an amorphous microstructure rather than a crystallized microstructure exhibits a good waterproof property.

The water resistance of multilayers was further studied. HfO_2_/SiO_2_ multilayers were prepared using the IAD process. After immersing samples in a hot water bath for several months, severe delamination was observed. The erosion morphologies of HfO_2/_SiO_2_ multilayers are shown in Figure S1 in the Supplementary Information. This result means that a multilayer does not possess a good waterproof property when one coating material is water resistant while another is not. Although water penetration perpendicular to the surface might be effectively blocked by dense amorphous SiO_2_ layers, water can diffuse along zigzag channels in HfO_2_ layers that are parallel to the surface, as illustrated in Fig. 3a1. Since there are always extrinsic defects in real multilayer coatings^19^, water molecules in HfO_2_ layers could reach and corrode the Nd:Glass substrate along defect-induced channels, which could be linked to the Nd:Glass surface.

**Fig. 3:**
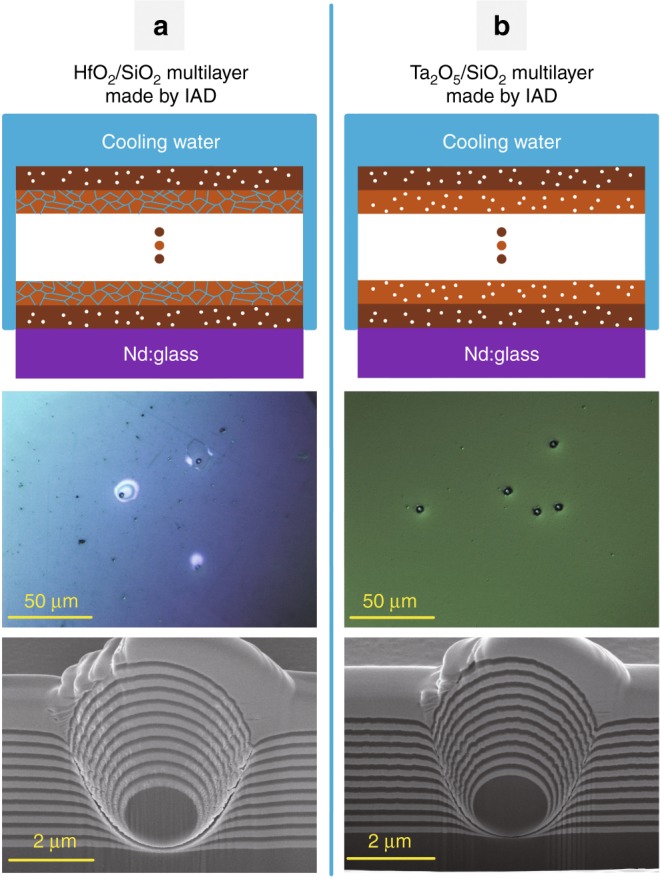
Schematic of water diffusion in nanopores; optical photographs; cross-sectional SEM images of HfO_2_/SiO_2_ and Ta_2_O_5_/SiO_2_ multilayers prepared by an IAD process

To verify the influence of defects, artificial nodules created from 2μm silica microspheres on Nd:Glass were tested in a hot water bath^20^. Figure 3a2 shows that blisters were observed at artificial nodules after serval hours of water bath heating. The cross-sectional scanning electron microscopy (SEM) image shown in Fig. 3a3 shows that large pores at nodular boundaries are open to the Nd:Glass surface, through which water can reach and erode the Nd:Glass. For comparison, artificial nodules in dense amorphous Ta_2_O_5_/SiO_2_ multilayers were also prepared using the IAD process. Because both Ta_2_O_5_ and SiO_2_ have a dense amorphous microstructure with closed nanopores, there are no channels for water diffusion along defect-induced channels, as shown in Fig. 3b. The Ta_2_O_5_/SiO_2_ multilayers did not peel off from the Nd:Glass substrate for the same water bath time. However, the LIDT for the Ta_2_O_5_/SiO_2_ multilayers is much lower than that for the HfO_2_/SiO_2_ multilayers because the bandgap of Ta_2_O_5_ is ~30% less than that of HfO_2_^21^. It is desirable to find novel coating materials to achieve an excellent waterproof property without sacrificing the LIDT.

Nanocomposites that dope SiO_2_ into HfO_2_ were synthesized using an ion-assisted co-evaporation process to obtain a dense amorphous microstructure, wider bandgap and higher LIDT. The higher the SiO_2_ concentration in Hf_*x*_Si_1−*x*_O_2_ nanocomposites, the better the resistance to crystallization. SiO_2_ (20%) in Hf_*x*_Si_1−*x*_O_2_ nanocomposites was not enough to suppress crystallization. Figure 4a shows that severe crystallization occurred after an initial amorphous growth phase. Hf_*x*_Si_1−*x*_O_2_ nanocomposites with a 30% SiO_2_ concentration or higher maintained a dense amorphous microstructure, as shown in Fig. 4b. From the aspects of coating design and electric-field control, it is optimal to use an amorphous Hf_*x*_Si_1−*x*_O_2_ nanocomposite, which has the highest refractive index. Therefore, an Hf_0.7_Si_0.3_O_2_ nanocomposite layer was used. The ultraviolet transmission spectra of the Hf_0.7_Si_0.3_O_2_, SiO_2_, HfO_2_ and Ta_2_O_5_ layers are compared in Fig. 4c. Together with the reflection spectrum, the bandgap of the Hf_0.7_Si_0.3_O_2_ nanocomposite layer was derived to be 6.4 eV using the Tauc algorithm^22^. The bandgap of this is much wider than that Ta_2_O_5_ and its LIDT should also be much higher.

**Fig. 4:**
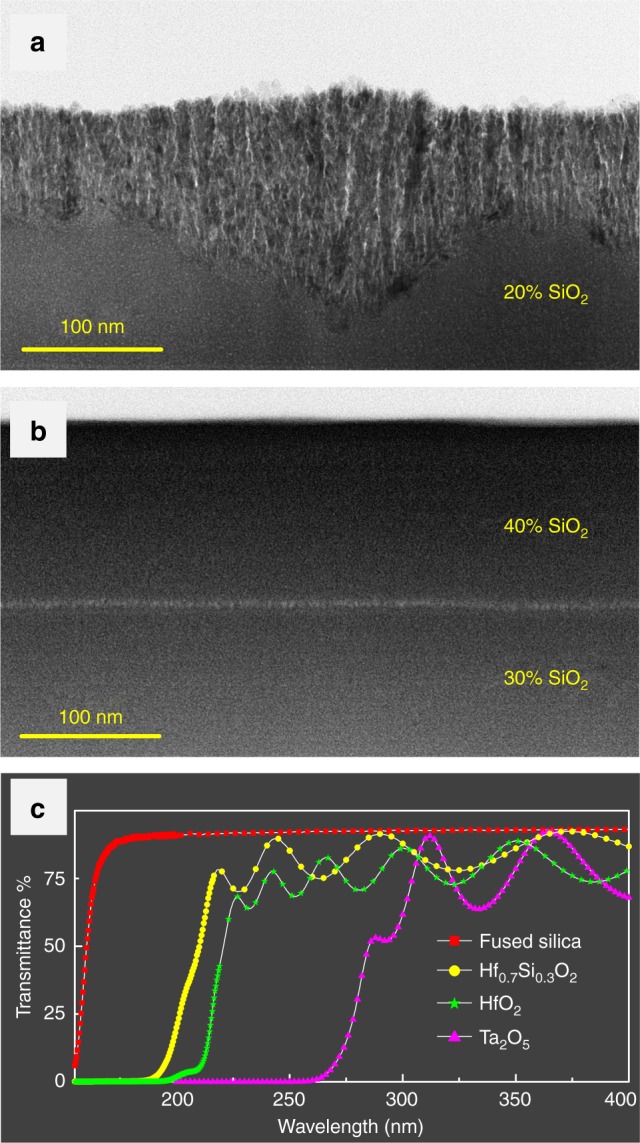
Comparisons among Hf_*x*_Si_1−*x*_O_2_ nanocomposite films and oxide films. Cross-sectional TEM images of Hf_*x*_Si_1−*x*_O_2_ nanocomposite layers with **a** 20% SiO_2_ concentration, **b** 30 and 40% SiO_2_ concentration. **c** Transmission spectra of single layers of Hf_0.7_Si_0.3_O_2_, SiO_2_, HfO_2_ and Ta_2_O_5_

Hf_0.7_Si_0.3_O_2_/SiO_2_ cavity mirrors were then prepared with a high reflectance at 1053 nm for s-polarization at the Brewster angle and a high transmittance at 802 nm for normal incidence^23^. Their LIDT was 34 ± 4 J/cm^2^ according to the method described by Borden et al.^24^, which is almost three times higher than the LIDT of Ta_2_O_5_/SiO_2_ cavity mirrors. This value is close to the LIDT value of ~42 ± 4 J/cm^2^ for a bare Nd:Glass substrate. By carefully controlling defects during the preparation of Nd:Glass substrates and coatings, Hf_0.7_Si_0.3_O_2_/SiO_2_ cavity mirrors can protect Nd:Glass from corrosion for over 1 year, even in a hot water bath.

Here, the influence of the pore characteristics on the waterproof property of single layers was determined. Dense amorphous coatings are water resistant because no channels exist for water diffusion. If one material is water resistant while another is not, the multilayer does not possess a waterproof property when extrinsic defects are present. Hf_*x*_Si_1−*x*_O_2_ nanocomposites were then synthesized to generate Hf_0.3_Si_0.7_O_2_/SiO_2_ cavity mirrors with the multifunctionality of an excellent waterproof property, high LIDT and good spectral performance.

## Materials and methods

### Preparation of laser coatings

SiO_2_, HfO_2_ and Ta_2_O_5_ single layers with a thickness of ~500 nm were deposited on Nd:Glass substrates using EBE and IAD processes. The deposition temperature was approximately 393 K, and the chamber was pumped down to a base pressure of 2.3 × 10^−4^ Pa. The IAD process employed was based on an RF-type ion source with a working condition of 600 V and 600 mA. Further details of the deposition process can be found in our previous paper^25^. Hf_*x*_Si_1−*x*_O_2_ nanocomposites were prepared using an ion-assisted co-evaporation process including annealing at 600 °C to reduce their absorption. A schematic of the ion-assisted co-evaporation process is shown in Figure S4 in the Supplementary Information. Further details of the process can be found in a previous paper^26^.

### Characterization of laser coatings

The water resistance of the prepared coatings was evaluated by immersing samples in a temperature-controlled water bath. Compared to a practical water-cooling situation, the temperature of the water bath was set to 90 °C to accelerate the failure process.

The microstructure of SiO_2_, HfO_2_ and Ta_2_O_5_ single layers was characterized using TEM. However, due to the low contrast of electron scattering between pores and the SiO_2_ matrix, it is difficult to clearly observe its microstructure. Based on the fact that the SiO_2_ coating has the same microstructure as a Ta_2_O_5_ coating^27^, a cross-sectional TEM image of a Ta_2_O_5_ coating was used instead to determine the microstructure of the SiO_2_ coating. For comparison, TEM images and electron diffraction patterns of SiO_2_ coatings are shown in Figures S2 and S3 in the Supplementary Information. The electron diffraction pattern of the crystallized part of the HfO_2_ coating is also shown in Figure S2 in the Supplementary Information. The microstructure of the artificial nodules in the HfO_2_/SiO_2_ and Ta_2_O_5_/SiO_2_ multilayers was determined by cutting them through the middle using focused ion beam technology. Then, the cross-sectional images shown in Fig. 3 were taken using SEM.

The concentration of SiO_2_ in Hf_*x*_Si_1−*x*_O_2_ nanocomposites was determined by fitting the refractive indices of the Hf_*x*_Si_1−*x*_O_2_ single layers using a Lorentz–Lorentz model^28^.

## Supplementary information

SUPPLEMENTAL MATERIAL
