# Supplementary material for: Waterproof coatings for high-power laser cavities
Source: Light Sci Appl. 2019 Jan 23;8:12. doi: 10.1038/s41377-018-0118-6 (PMC6342822; doi:10.1038/s41377-018-0118-6)
Supplement: Supplementary file 1 — SUPPLEMENTAL MATERIAL [file 41377_2018_118_MOESM1_ESM.docx]

**Supplementary Information**

Waterproof coatings for high power laser cavities

Xinbin Cheng, Siyu Dong, Zhi Song, Sebastian Paschel, Istvan Balasa, Detlev Ristau, Zhanshan Wang*

The erosion morphologies of HfO_2_/SiO_2_ multilayer that was prepared by an IAD process were recorded using a camera and optical microscopy.


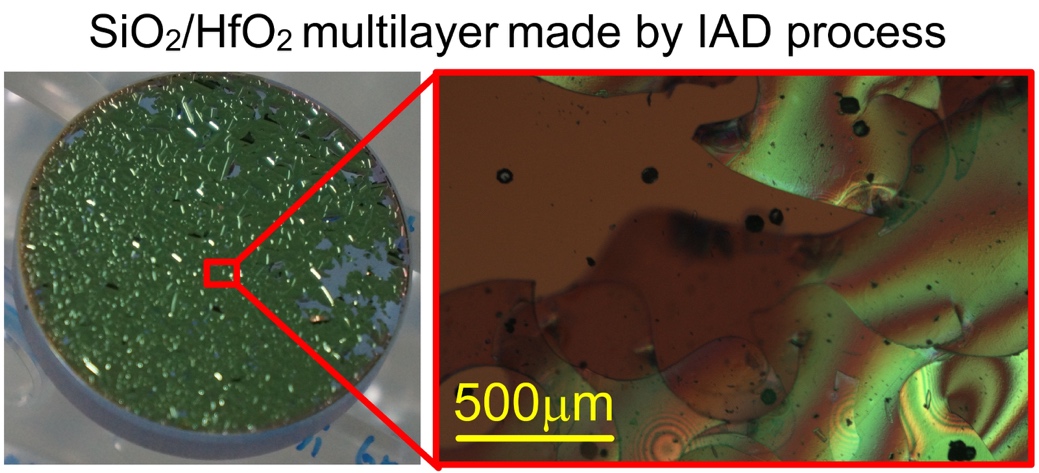


**Figure S1.** The erosion morphologies of the HfO_2_/SiO_2_ multilayer

Cross-sectional TEM images of SiO_2_ coatings prepared using EBE and IAD processes. Due to the low contrast of electron scattering between pores and SiO_2_ matrix, it is difficult to resolve the pores distribution and to evaluate the difference of two SiO_2_ coatings that were prepared using EBE and IAD processes.


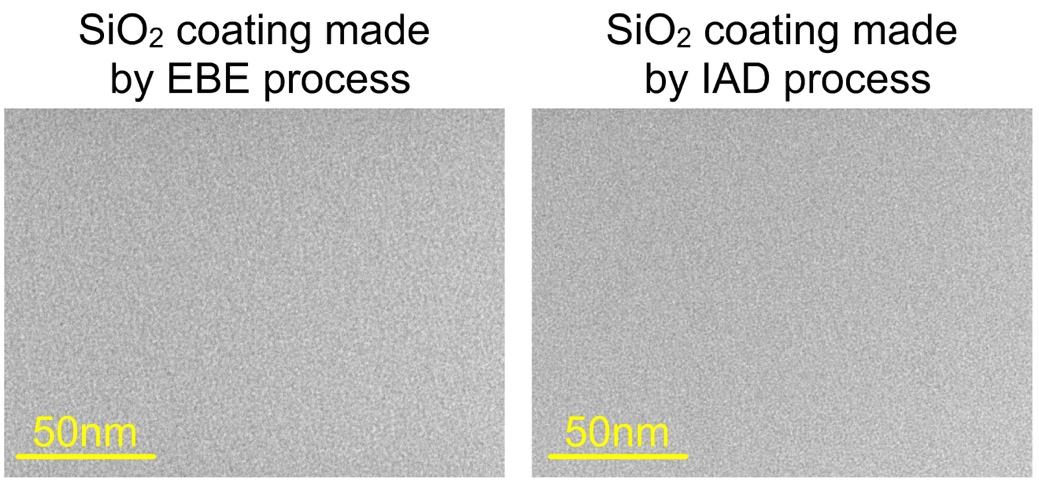


**Figure S2.** TEM images of SiO_2_ coatings prepared using EBE and IAD processes.

Electron diffraction patterns of four types of coatings, they can reflect whether the coating has an amorphous or polycrystalline microstructure. The amorphous halo is clearly visible in both two SiO_2_ coatings. For the HfO_2_ coating made by EBE process, its selected area electron diffraction (SAED) pattern shows the disordered crystalline diffraction spots, which is consistent with the results shown in TEM image of Figure 1b that there is a transition from an amorphous microstructure to a polycrystalline microstructure as the coating grows thicker. In the SAED pattern of HfO_2_ coating made by IAD process, the feature diffraction rings are obvious and assigned to the (1 1 1), ( 1 1) and (0 2 0) lattice planes, which confirms that the coating is composed of uniform polycrystalline HfO_2_.


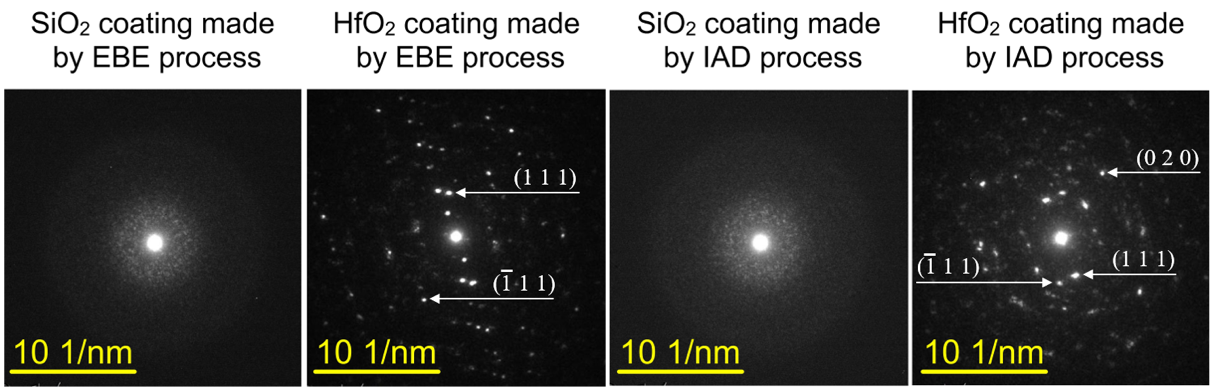


**Figure S3.** Electron diffraction patterns of SiO_2_ and HfO_2_ coatings prepared using EBE and IAD processes.

A schematic of ion-assisted co-evaporation technique is shown in **Figure S3**. The Hf_x_Si_1-x_O_2_ nanocomposite film was deposited on the substrates by co-evaporating HfO_2_ and SiO_2_. The two quartz crystal monitors were used to monitor the deposition rates of both materials, and the two programmable masks were used to control them. The refractive indices of the Hf_x_Si_1-x_O_2_ nanocomposites can be varied in a wide range with different relative deposition rates of both materials.


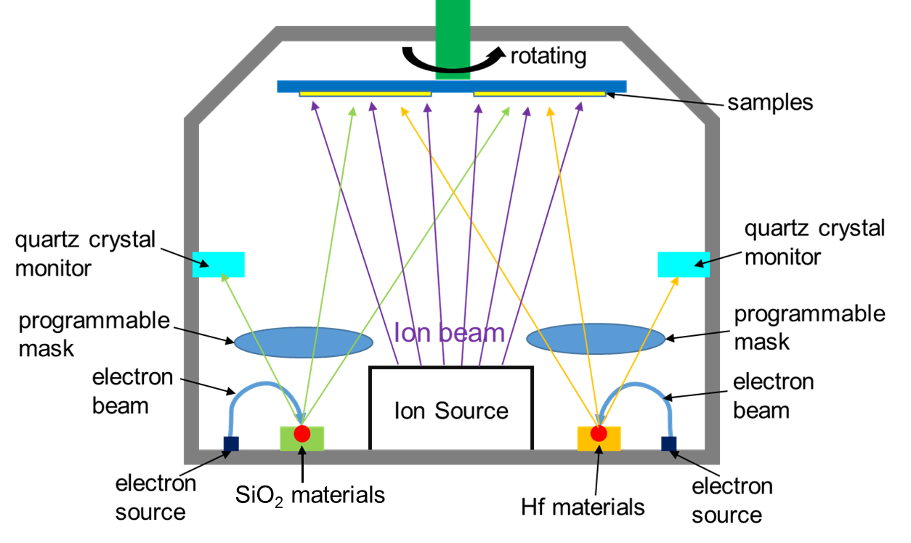


**Figure S4.** Schematic of ion-assisted co-evaporation process
